# Molecular mechanisms of the juvenile form of Batten disease: important role of MAPK signaling pathways (ERK1/ERK2, JNK and p38) in pathogenesis of the malady

**DOI:** 10.1186/s13062-018-0212-y

**Published:** 2018-09-25

**Authors:** Elena K. Shematorova, Dmitry G. Shpakovski, Anna D. Chernysheva, George V. Shpakovski

**Affiliations:** Shemyakin-Ovchinnikov Institute of Bioorganic Chemistry, Russian Academy of Sciences, Miklukho-Maklaya 16/10, GSP-7, 117997 Moscow, Russia

**Keywords:** Juvenile neuronal ceroid lipofuscinosis (Batten disease), CLN3, Molecular chaperone, ATP1A1, SRC, EGFR, MAPK signaling

## Abstract

**Background:**

Mutations in the *CLN3* gene lead to so far an incurable juvenile-onset neuronal ceroid lipofuscinosis (JNCL) or Batten disease that starts at the age of 4–6 years with a progressive retinopathy leading to blindness. Motor disturbances, epilepsy and dementia manifest during several following years. Most JNCL patients carry the same 1.02-kb deletion in the *CLN3* gene, encoding an unusual transmembrane protein, CLN3 or battenin.

**Results:**

Based on data of genome-wide expression profiling in *CLN3* patients with different rate of the disease progression [Mol. Med., 2011, 17: 1253–1261] and our bioinformatic analysis of battenin protein-protein interactions in neurons we propose that CLN3 can function as a molecular chaperone for some plasma membrane proteins, being crucially important for their correct folding in endoplasmic reticulum. Changes in spatial structure of these membrane proteins lead to transactivation of the located nearby receptors. Particularly, CLN3 interacts with a subunit of Na/K ATPase ATP1A1 which changes its conformation and activates the adjacent epidermal growth factor receptor (EGFR). As a result, a large amount of erroneously activated EGFR generates MAPK signal cascades (ERK1/ERK2, JNKs and p38) from cell surface eventually causing neurons’ death.

**Conclusions:**

Molecular mechanism of the juvenile form of Batten disease (JNCL), which is based on the excessive activation of signaling cascades in a time of the radical increase of neuronal membranes’ area in the growing brain, have been proposed and substantiated. The primary cause of this phenomenon is the defective function of the CLN3 protein that could not act properly as molecular chaperone for some plasma membrane proteins in the endoplasmic reticulum. The incorrect three-dimensional structure of at least one such protein, ATP1A1, leads to unregulated spontaneous and repetitive activation of the SRC kinase that transactivates EGFR with the subsequent uncontrolled launch of various MAPK cascades. Possible ways of treatment of patients with JNCL have been suggested.

**Reviewers:**

This article was reviewed by Konstantinos Lefkimmiatis, Eugene Koonin and Vladimir Poroikov.

## Background

The neuronal ceroid lipofuscinoses (NCLs) are the most common inherited neurodegenerative diseases mainly affecting children, with a combined incidence estimated to be as high as 1 in 12,500 [1]. Clinical features include progressive visual loss leading to complete blindness, motor and mental decline (up to ataxia and dementia), epilepsy, and premature death. The NCLs are named for the “ceroid lipofuscin” lysosomal storage material observed in neurons and many other cell types, including those outside of central nervous system (CNS). Mutations in at least eight genes (*CLN1-CLN3*, *CLN5-CLN8* and *CLN10*) have been identified for these pediatric disorders. Despite excessive in vitro and in vivo studies, the precise functions of the CLN proteins and the NCLs’ disease mechanisms remain elusive [2]. Juvenile form of Batten disease is the most common variety of different NCLs. Therefore, it is not surprising that the main efforts in studies of neuronal lipofuscinoses were aimed at clarifying the molecular mechanisms of this particular disease.

### Important role of MAPK signaling pathways in molecular mechanisms of the juvenile form of Batten disease

Recent studies have shown that careful investigation of genome-wide gene expression changes, including consideration of both the types of affected tissues and systems and the classification of patients according to the rate of disease progression, plays an important role in establishing the causes of complex human diseases. For instance, it was the separation of patients into groups according to the rate of the disease progression that recently allowed *M. Davis* and his colleagues to show that chronic fatigue syndrome (CFS), also called myalgic encephalomyelitis (ME) (ME/CFS), is associated with the inflammatory process [3].

The same strategy has been well implemented earlier in the work of *Lebrun* et al. (2011) for exploring the genes influencing the course of JNCL [4]. Twenty five patients, who are homozygous for the characteristic deletion of 1.02 kb in *CLN3* gene, were divided into 3 groups according to the speed of the disease progression (slow, average and rapid). In attempts to find meaningful changes in gene expression patterns correlated with the clinical variability of the Batten disease, the genome-wild microarray analysis was performed in lymphocytes isolated from the blood of 8 patients with different rates of the disease development. In conclusion, 18 significantly differently expressed genes were identified and subdivided in two main categories: 5 biomarkers (genes that are dysregulated by a similar way in all three groups) and 13 modifiers (genes that were upregulated in patients with rapid disease progression and downregulated in patients with slow disease progression or vice versa).

Surprisingly, genes involved in biogenesis of lysosomes have not been found in this search, but genes encoding membrane receptors/coreceptors and/or regulating signal cascades were clearly represented. Taking this notion into account and further analyzing and critically interpreting the results of the work [4], we came to the conclusion that most (at least 14) genes with significantly altered levels of expression in CLN3 patients could be divided into the following functional groups shown in Table 1. The data presented in the Table 1 indicate that mild form of the disease is characterized by similar behavior of genes within the same functional class. Indeed, the plasma membrane receptor/coreceptor genes (Class I), genes encoding proteins involved in the signaling cascade transduction (Class II), and genes coding for components of the transcription apparatus (Class IV) were all greatly suppressed in a mild form of Batten disease.

**Table 1:** Classification of deregulated genes found in patients with Batten disease [4]*

Functional classes	Genes	Functions of encoded protein	Type of action	Mild variety of JNCL	Classic JNCL
I. Genes coding for receptors and coreceptors	*GPR109B*	Receptor of caprilic acid, GPCR, involved in metabolism of lipids and cholesterol.	Modifier	Down	Up
	*FCGR2B/* *FCGR2C* *(CD32)*	Low affinity receptor for the Fc region of immunoglobulin gamma complexes.	Modifier	Down	Up
	*MS4A1(CD20)*	Member of the membrane-spanning 4A gene family. MS4A proteins form signaling complexes with other surface membrane molecules that modulate downstream biochemical signals [26].	Modifier	Down	Up
II. Genes encoding proteins involved in signal transduction	*BLK*	Src-like tyrosine kinase.	Modifier	Down	Up
	*RAPGEF1*	Guanine nucleotide exchange factor activating several members of the Ras family of GTPases.	Modifier	Down	Up
	*CDC42SE2*	CDC42 small effector 2 involved in signal transduction from B-lymphocyte receptors, GPCRs and receptors of tyrosine kinases.	Biomarker	Down	Down
	*MARCKS*	Participates in protein kinase C (PKC)-MARCKS-PIP2-PI3K-PIP3 cascade [27].	Modifier	Down	Up
	*BACE2*	APP cleavage and transcription activation through AICD.	Modifier	Down	Up
III. Genes coding for proteins that impair signaling cascades	*RGS1*	Protein from family of negative regulators of GPCR signaling pathways.	Biomarker	Up	Up
	*DUSP2*	Threonine/tyrosine phosphatase localizing in the nucleus and specifically dephosphorylating MAP superfamily kinases.	Biomarker	Up	Up
IV. Genes encoding transcription factors and components of the RNA polymerase II (RNAPII)	*KLF6*	A member of the Kruppel-like family of transcription factors.	Modifier	Down	Up
	*SPIB*	A transcriptional factor (TF) that binds to the PU-box (5′-GAGGAA-3′) and belongs to ETS family of TFs.	Modifier	Down	Up
	*POLR2J2*	A minor isoform of the RNA polymerase II subunit hRPB11	Biomarker	Down	Down
	*ZNF718*	Zn-finger protein that may be involved in transcriptional regulation.	Modifier	Down	Up

*Functional grouping of the 4 remaining genes (*CLLU1*, *SOLO*, *LOC283663* and *PARP15*) is still not clear and needs additional clarification.

At the same time, expression of genes encoding proteins that inhibit signaling cascades (Class III) was strongly activated. Apparently, these changes in gene expression represent adaptive cellular responses to the CLN3 protein dysfunction that makes the symptoms of the disease less severe.

Thus, presumably in lymphocytes of patients with JNCL there are changes in gene expression aimed at the suppression of certain signaling cascades. These signal cascades are activated by lymphocyte-specific receptors (IgG, MS4A1, GPR109B) characteristic for cells of the immune system.

**Fig. 1:**
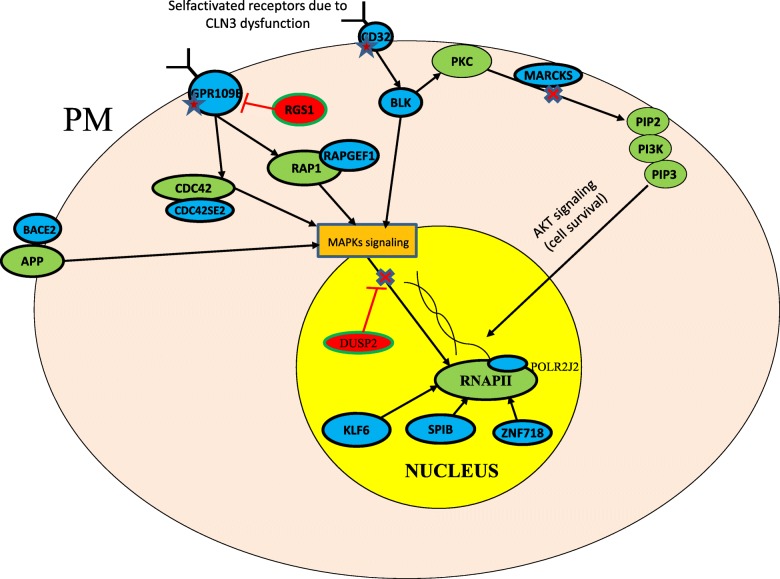
The scheme of interaction of products of disordered genes in patients with Batten disease, found in the article of Lebrun et al. [4]. Blue ovals depict proteins whose genes are suppressed in patients with the slow progression form of the disease and red ovals denote proteins whose genes are overexpressed; green ovals represent proteins, functionally interacting with protein products of disordered genes

Scheme of functional interactions of protein products of the aforementioned genes from 4 classes is depicted on the Fig. 1. *DUSP2* gene, which is considerably upregulated in all patients with Batten disease, plays an important role in eradiation of the erroneous accessory signals coming to the cell nucleus. As it was clearly shown in [4], all CLN3 patients overproduced DUSP2 dual specificity phosphatase which dephosphorylates both extracellular signal-regulated (ERKs) and stress-activated (p38 and JNKs) protein kinases and acts as an antagonist of associated MAPK signaling cascades. We assume that self-activation of receptors occurs in the affected cells, and the simplest explanation for this phenomenon can be a change in the secondary-tertiary structure of these molecules caused by CLN3 dysfunction. Earlier, it was established that CLN3 protein is localized mostly in the endoplasmic reticulum and lysosomes, and in the brain also at the plasma membrane [1, 2]. This led us to think that CLN3 could function as a kind of molecular chaperone for the aforementioned plasma membrane receptor proteins in lymphocytes and is crucially important for their correct folding. Incorrect folding of the plasma membrane proteins leads to initiation of signaling cascades from the cell surface.

### Na+/K+ ion channels ATPase ATP1A1 interacts with CLN3 and activates signal cascades

In order to understand which receptors can be a cause of the signaling cascades’ activation in neurons, we have analyzed the interaction of CLN3 with proteins of the plasma membrane, using the available databases of protein-protein interactions BioGRID (http://www.thebiogrid.org), MINT (http://mint.bio.uniroma2.it), and STRING (http://string.embl.de). Surprisingly, the true receptors that can trigger a signaling cascade from the membrane surface were not found. However, it was revealed and not escaped our notice that CLN3 interacts with such proteins of ion channels as Na^+^/K^+^ ATPase ATP1A1 (α1) subunit [5, 6].

The α1 (first catalytic) subunit of Na/K-ATPase possesses both pumping and signaling functions. This protein, ATP1A1, might interact with non-receptor tyrosine kinase SRC and regulates its activity in a conformation-dependent manner [7]. It was also shown that cardiac glycoside ouabain [7], hydrogen peroxide [8] and ammonium chloride [9] might provide conformational change in Na/K-ATPase ATP1A1 that leads to SRC and subsequent EGFR activation. So, conformational change in α1 Na/K-ATPase and its activation of SRC can be easily transmitted to MAPK cascades.

Indeed, recent works showed that activation of SRC was essential for ouabain-induced activation of ERK1/ERK2 and p38 [10]. MAPK (mitosis activated protein kinases) ERK1/ERK2 are responsible for the growth and proliferation of cells and participate in the activation of genes that provide the synthesis of growth factors and mitogens. However excessive, uncontrolled activation of these signaling pathways may lead to overproduction of some proteins and their toxicity, which could be the cause of the cells death. The p38 and JUN pathways have also been implicated in stress-induced signaling leading to apoptosis [11].

Thus, we suggest that CLN3 is involved in the correct formation of the АТР1А1 spatial structure in neurons. Mutations or truncations of this protein lead to a shift of ATP1A1 to the signaling conformation and activation of the EGFR through SRC kinase. Intensive expansion of the plasma membrane area in neurons, which is particularly important in the early period of human life, leads to the substantial increase of ion channels with ATP1A1 in them, and, consequently, increases the number of activated signal cascades sent to the cell nucleus. When neurons are unable to suppress excessive signaling cascades, their death is imminent.

### The search for possible additional neural membrane CLN3 partners activating signal cascades

Using available databases of protein-protein interactions (BioGRID, MINT and STRING), we performed an additional search where we looked for the proteins that matched to the following criteria:

1. The protein interacts with CLN3.
2. The interaction has been detected in the neuronal cells.
3. The function of this protein is associated with the plasma membrane.
4. The protein interacts with some receptors participating in signal cascades activation or inhibition.
5. The protein expression in the brain has to be high (according to Human Protein Atlas http://www.proteinatlas.org).

This additional search has revealed only two proteins that meet all our criteria (Table 2). It should be mentioned that both proteins (KCNIP3 and TFRC) interact with receptors which are able to activate MAPKs cascades [12–14]. Only additional experiments could indicate that these proteins by changing their spatial structure can transactivate nearby receptors.

**Table 2:** Protein partners of CLN3 matching to the aforementioned search criteria

Protein partner of CLN3	Function	Interaction with receptor complexes	Putative signal cascade activation	Level of protein expression in the brain
KCNIP3 [28]	Voltage-gated potassium (Kv) channel-interacting protein	IGF1R [12]	MAPKs	High
TFRC [6]	A cell surface receptor necessary for cellular iron uptake by the process of receptor-mediated endocytosis	EGFR [13], NTRK1 [14]	MAPKs	High

Excessive receptor activation can be the cause of other neurodegenerative diseases. For example, it was shown that the IGF1R and RAS–MAPK–MSK1 signaling pathway could be a cause of spinocerebellar ataxia type 1 (SCA1) development [15]. Several antagonists for adenosine receptors have been able to reverse cognitive impairments in animal models of Huntington’s disease [16]. A new drug (Nouriast R) that works by blocking the activation of A2A receptors has now been approved for the treatment of Parkinson’s disease [17, 18].

### NCL proteins possess a common function in ER, their dysfunction could lead to improper functioning of EGFR and to excessive MAPK cascades activation

NCL proteins differ in their functions and their final intracellular localization. These polypeptides have been localized mostly in lysosomes (CLN1, CLN2, CLN3, CLN5, CLN7 and CLN10) but also in the endoplasmic reticulum (CLN3, CLN6, CLN8). CLN1 and CLN3 have also been detected on the plasma membrane [1]. Some of them are soluble proteins (CLN1, CLN2, CLN5, CLN10), other like CLN3, CLN6, CLN7 and CLN8 are transmembrane molecules.

Nevertheless, classical NCL protein mutations show an autosomal recessive mode of inheritance, and are classified according to the ages of clinical disease’ onset such as congenital (CLN10), infantile (CLN1), late infantile (CLN5, CLN6, CLN7 and CLN8) and juvenile (CLN3). They share clinical features such as progressive loss of vision as well as mental and motor deterioration, epileptic seizures, and eventually premature death.

**Fig. 2:**
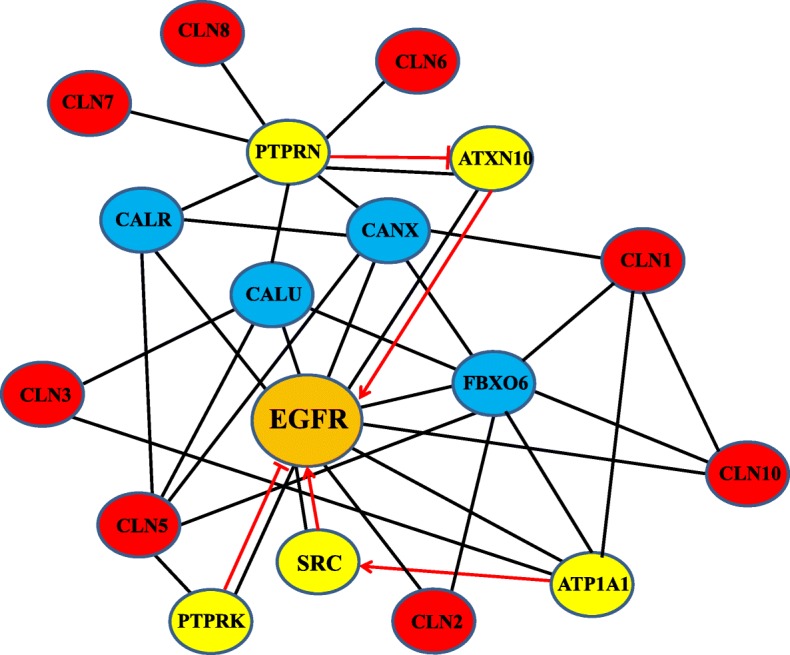
CLN1, CLN2, CLN3, CLN5, CLN6, CLN7, CLN8 and CLN10 proteins can be connected in a single protein network. The protein network was generated by using software tool *esyN* (http://www.esyn.org/) integrated with major databases. Red ovals represent NCL proteins, blue ovals – protein residents of the endoplasmic reticulum, yellow ovals – plasma membrane proteins. Red lines with perpendicular bar at their ends and red arrows indicate negative (inhibitory) or positive (activatory) effects of the corresponding protein interactions

CLN5 co-immunoprecipitation with five other NCL proteins (CLN1, CLN2, CLN3, CLN6 and CLN8) has been shown in the work [19]. These interactions can occur only in the endoplasmic reticulum. In addition, our bioinformatics analysis (by using BioGRID, MINT and STRING) indicates that all 8 NCL proteins can be connected in a single protein network (Fig. 2).

They closely interact with calcium-binding proteins like calnexin (CANX), calumenin (CALU) and calreticulin (CALR) which are localized in the endoplasmic reticulum and involved in protein folding and sorting, as well as with FBXO6 protein, a subunit of chaperonin TRiC/CCT complex which is responsible for the correct folding of some glycoproteins.

We conducted an additional analysis of protein-protein interactions (by using BioGRID, MINT and STRING) and confirmed that all NCL polypeptides either interact directly with EGFR or contact with membrane proteins modulating EGFR activity. Thus, mutations in other NCL proteins, which together with their interacting partners CANX, CALR, CALU and FBXO6 can be considered as some kind of chaperones for these membrane proteins, leads to improper functioning of EGFR and, as a consequence, to uncontrolled activation of MAPK cascades (Fig. 2).

### Testing of the hypotheses and possible implication for treatment of patients with Batten disease (JNCL)

To test our hypothesis it is necessary to assess the level of activation of the Src family of non-receptor tyrosine kinases (SFKs), and components of MAPKs cascades in cell cultures obtained from patients affected by Batten disease (JNCL), as well as in the brain cells of mice with different types of deletion in the *CLN3* gene [20–22]. If, in the process of testing our hypothesis, increased levels of activated SFKs (e.g. SRC) will be found, a way to treat the juvenile form of Batten disease could be to use the SFK inhibitors.

Dasatinib (Bristol Myers Squibb) is a selective and potent SFK inhibitor with a SRC, FYN and LCK potency. This drug is approved for the treatment of chronic myeloid leukemia (CML), and has shown some promise in modulating microglial activation and improving memory function in transgenic Alzheimer’s disease (AD) mice [23]. A clinical trial in AD patients with another SFK inhibitor, saracatinib, is currently under way [23].

The proposed model of pathological changes in the neurons of patients with JNCL (Batten disease) also suggests the possibility of using inhibitors of EGFR [24] and ERK1/2, JNKs and p38 signaling [25].

## Conclusions

A molecular mechanism of the juvenile form of Batten disease (JNCL), which is based on the excessive activation of signaling cascades at the time of the radical increase of neuronal membranes’ area in the growing brain, has been proposed and discussed. It is proposed that the primary cause of this phenomenon is the defective function of the CLN3 protein that does not act properly as molecular chaperone for some plasma membrane proteins in the endoplasmic reticulum. The incorrect three-dimensional (spatial) structure of at least one such protein, ATP1A1, leads to unregulated spontaneous and repetitive activation of the SRC kinase that transactivates EGFR with the subsequent uncontrolled launch of various MAPK cascades. Possible ways of treatment of patients with JNCL have been suggested, which may slow down, significantly delay or even (at least temporally) stop the development of this so far incurable devastating disease.

## Reviewers’ comments

We are very grateful to all the Reviewers for their thoughtful and valuable comments. Here are our answers and/or explanations regarding their specific comments.

### Reviewer’s report 1: Konstantinos Lefkimmiatis, Italian National Research Council (CNR), Italy

Batten disease is an incurable and fatal disorder that in most cases is connected to a mutation of the *CLN3* gene. Despite the well-characterised genetic defect a molecular explanation of how *CLN3*-mutations affect neuronal physiology is still missing. In this manuscript Shematorova and colleagues perform a series of bioinformatics analysis using publicly available databases. The data obtained are interpreted and lead to the formulation of the hypothesis that deregulation of MAPK pathways may be involved in CLN3-dependent Batten disease. The data produced are well thought-through and support the author’s thesis, nevertheless the manuscript could benefit from some editing aiming to better showcase the connection between the different sessions. I would suggest to the authors to more extensively develop the following parts: - In the first session “Important role of MAPK signaling pathways in molecular mechanisms of the juvenile form of Batten disease” the reasoning behind the authors choice to assign each gene in the 4 classes should be made more clear. - In the same session: While it can be implied that over expression of *DUSP2* supports well the idea of non-canonical MAPK activation this is not very clear, this connection should be discussed more expensively.

Authors’ response: *Following the Reviewer suggestion to extend explanation for our classification of the genes affected on 4 different classes and clarification of the possible role of DUSP2 overexpression, we have added a new* Fig. 1 *showing the possible scheme of the involvement of the aforementioned genes in the excessive MAPK cascades activation.*

- In the criteria of the CLN3 partner selection the authors set 5 different filters. The 4th filter limits their search to proteins that interact with some (??) receptors participating “in signal cascade activation”. Do the authors exclude receptors that participate in the signal cascade inhibition? (e.g. Gi-coupled receptors?). This has to be clarified. - While the information provided is interesting, the session where the connection between NCL proteins and ER is discussed does not contribute to the MAPK-based hypothesis of the manuscript. I wonder if this part is to be better integrated or eliminated from the final version of the manuscript. While the manuscript is well written, there are parts that would benefit from editing to simplify.

Authors’ response: *By adding the option “search for proteins that participate in the signal cascade inhibition” to the filters, we conducted an additional analysis of protein-protein interactions and confirmed that all NCL polypeptides either interact directly with EGFR or contact membrane proteins modulating EGFR activity. Thus, mutations in other NCL proteins, which together with their interacting partners CANX, CALR, CALU and FBXO6 can be considered as some kind of chaperones of these membrane proteins, apparently lead to improper functioning of EGFR and, as a consequence, to uncontrolled activation of MAPK cascades (*Fig. 2*).*

- In the new version of the manuscript the authors made an effort to answer to the issues raised and, at least to some extent, made the basis of their hypothesis clearer. It is important to point out that this is a hypothesis, based on publicly available datasets that contain information not always extensively validated, thus only experimental work (if engaged) will confirm their theory (or not).

Authors’ response: *We agree with the Reviewer that experimental confirmation of the proposed hypothesis should be obtained in the course of further research.*

- Although the manuscript benefited from editing of the language, in some parts the writing remains odd.

Authors’ response: *Following the Reviewer suggestion, the manuscript was edited by qualified native English speaker (please, see the Acknowledgement section).*

### Reviewer’s report 2: Eugene Koonin, NCBI, NLM, NIH, USA

The authors of this Hypothesis paper analyze the available data on gene differential expression in Batten disease as well as protein-protein interaction, and on the basis of such analysis, come up with a hypothesis explaining some aspects of Batten disease pathogenesis, and even make suggestions regarding possible therapeutic interventions. The attempt to make the most of the available “omic” data to understand disease mechanism is commendable. However, I find the evidence (if it can be called that) for a membrane chaperone function of CLN3, which is the key point of the hypothesis, to be tenuous at best. Actually, based on my own reading of the literature, a more general defect in lysosomal biogenesis seems to be the most likely consequence of disease-associated mutations in CLN3. Of course, the authors are entitled to their speculation, which does not directly contradict the available data. However, I do not find the proposed mechanism to be either substantially supported or parsimonious. Accordingly, I think the authors should re-examine their argument carefully, tone down some of the predictions, and perhaps, come with alternatives.

Authors’ response: *Following the Referee’s suggestion, we modified some of our statements and predictions. This, however, did not change the basic idea of our work on the importance of signal transmission and particularly MAPK cascades signaling in the developing of the main neurological symptoms of the malady which we consider as an alternative to the ‘lysosome biogenesis’ hypotheses. Although originally Batten disease was classified as one of the lysosomal storage diseases and many studies were aimed at investigating this particular aspect of the disease, these studies did not lead to a serious breakthrough in understanding the molecular mechanisms of this disease and/or finding potential ways to treat it. Moreover, one can think of several reasons that the main mechanism of Batten disease as a neurodegenerative disorder cannot be associated with a general defect in lysosomal biogenesis.*

*Indeed, the mouse model showed that the harmful impact of the common JNCL mutation on the CNS was not well correlated with membrane deposition* per se*, suggesting instead a special battenin activity that is essential for the survival of CNS neurons. This apparent paradox suggests that membrane deposits themselves may not be a toxic entity. Instead, the disease process may evolve a special battenin-dependent pathway that is essential for neuronal cell survival, consistent with battenin in neurons* [22]*.*

*It is also worth noting that in neurons most susceptible to pathological changes in Batten disease, CLN3 is detected mainly near the plasma membrane of the cell soma, as well as in the neural extensions and synaptic terminals (Luiro K.* et al.*, 2001). The hyperactivation of the immune system and neuroglia are two symptoms common to all patients with Batten disease. These phenomena can be explained very easily by activation of excessive signal cascades, which stimulate synthesis of excessive amounts of antibodies and other components of inflammatory reactions* [1, 2]*.*

- The English of the manuscript requires considerable attention. It is intelligible but, in many places, awkward. Editing by a native English speaker or equivalently qualified colleague is highly desirable.

Authors’ response: *Thank you for a suggestion. The manuscript was edited by a native English speaker.*

### Reviewer’s report 3: Vladimir Poroikov, Institute of Biomedical Chemistry of Rus. Acad. Med. Sci, Russia

The main emphasis of this work is the hypothesis that CLN3 protein has a chaperone function for some membrane proteins. Thus, the mutations in CLN3 gene led to the conformational changes of those proteins may cause the subsequent deviations in signaling cascades, which is considered as a molecular mechanism of juvenile Batten disease. Bioinformatics analysis of known protein-protein interactions allowed identifying the Na/K ATPase ATP1A1 as a potential victim of CLN3 malfunction. In turn, the conformational change in ATP1A1 leads to the stimulation of non-receptor tyrosine kinase SRC and excessive activation of MAPK signaling pathway. Based on the suggestions listed above, the authors proposed to use SRC kinase inhibitors (e.g., Dasatinib or Saracatinib) for treatment of juvenile Batten disease. Despite the absence of direct evidence supported the authors’ hypothesis, the manuscript could be recommended for the publication because it helps to determine the ways for experimental studies of the Batten disease mechanisms and to identify new pharmacological targets for its treatment. However, some additional comments must be considered by the authors before the publication.

It would be great if the authors could more clearly present the pieces of evidence why they suggest that change of Na/K ATPase ATP1A1 conformation interacting with the mutant form of the CLN3 protein is due to the breach of CLN3 chaperone function.

Authors’ response: *The work of the laboratory Xie Z.* [7] *clearly showed that Na/K ATPase ATP1A1 also has an important signaling function* via *EGFR activation. Other authors* [5, 6] *not only proved the interaction between proteins CLN3 and Na/K ATPase ATP1A1, but also showed that functions of ATP1A1 as a pump were not significantly affected in patients with Batten disease. So most likely in this case, it seems that the signaling function is affected, as we have postulated in our hypothesis. Of course, experimental confirmation of this idea should be obtained in the course of further research.*

2. Analysis of the available information about recent studies in the field of juvenile Batten disease has shown that several ways for its treatment are investigated now. From my point of view, these studies should be mentioned in the paper with the discussion of advantages and pitfalls of those methods comparing to the proposed therapeutic approach. In particular, I mean the application of phosphodiesterase 4 inhibitors (Aldrich A. et al. Ann Neurol., 2016, 80 (6): 909–923), self-complementary adeno-associated viral constructs, that express the human gene CLN3 (WO 2016100575), antisense oligonucleotides (WO 2015084884).

Authors’ response: *All studies mentioned by the Reviewer were conducted in a mouse model of Batten disease. The symptoms of this disease in mice do not correspond to the symptoms of the disease in humans. For example, the introduction of 1,02 deletion in the mouse cln3 gene causes the retina degeneration only in the second half of the animals’ life, and all the other symptoms are manifested in a very mild form. Therefore, the proposed inhibitors should be tested on humans. Although treatment methods with use of the adeno-associated viral constructs or antisense oligonucleotides were patented in 2015–2016, we did not find any reports in the medical literature that they were successful in treating patients with the known versions of Batten disease.*

3. Why do the authors not consider the ATP1A1 inhibitors as potential remedies for the treatment of juvenile Batten disease? There are such pharmacological agents in Phase II clinical trials.

Authors’ response: *These inhibitors are aimed at blocking the function of the ATP1A1 ATPase as a pump. Since it was shown that the function of Na+/K+ ions exchange was not affected in patients with Batten disease, the proposed inhibition would not be effective.*

4. From my point of view, Fig. 1 must be extended to cover the SRC kinases discussed in the text of the manuscript. Also, it would be nice to reflect in this figure the effects of protein-protein interactions (activation/inhibition).

Authors’ response: *We have added the SRC kinase in* Fig. 2 *where we also show the effects of some protein-protein interactions (activation/inhibition).*

In the search for additional neural membrane CLN partners the authors mentioned that they used available databases of protein-protein interactions. If these databases are the same that were mentioned above (MINT, BioGRID, and STRING)?

Authors’ response: *Yes.*

Some terms (NCL, ER, etc.) should be added to the list of abbreviations.

Authors’ response: *Done.*

## Abbreviations

AICD: APP intracellular domain
ATP1A1: ATPase Na+/K+ transporting subunit alpha 1
ATXN10: Ataxin 10, involved in spinocerebellar ataxia, type 10
CALR: Calreticulin
CALU: Calumenin
CANX: Calnexin
CLN3: Ceroid lipofuscinosis, neuronal 3
CNS: Central nervous system
DUSP2: Dual specificity phosphatase 2
EGFR: Epidermal growth factor receptor
ER: Endoplasmic reticulum
ERK: Extracellular signal-regulated kinase
FBXO6: Subunit of chaperonin TRiC/CCT complex
FYN: FYN proto-oncogene, Src family tyrosine kinase
GPCR: G-protein coupled receptor
JNCL: Juvenile-onset neuronal ceroid lipofuscinosis
JNK: c-Jun NH2-terminal kinase
LCK: LCK proto-oncogene, Src family tyrosine kinase
MAPK: Mitogen-activated protein kinase
ME/CFS: Myalgic encephalomyelitis / chronic fatigue syndrome
NCL: Neuronal ceroid lipofuscinosis
p38: Mitogen-activated protein kinases 11- 14
PKC: Protein kinase C
PM: Plasma membrane
PTPRK: Protein tyrosine phosphatase, receptor type K
PTPRN: Protein tyrosine phosphatase, receptor type N
RNAPII: DNA-dependent RNA polymerase II
SFK: Src family of non-receptor tyrosine kinases (SFKs)
SRC: SRC proto-oncogene, non-receptor tyrosine kinase
TF: Transcription factor
